# Bayesian detection of periodic mRNA time profiles without use of training examples

**DOI:** 10.1186/1471-2105-7-63

**Published:** 2006-02-09

**Authors:** Claes R Andersson, Anders Isaksson, Mats G Gustafsson

**Affiliations:** 1 The Linnaeus Centre for Bioinformatics, BMC, Uppsala University, Box 598, S-751 24 Uppsala, Sweden; 2 Department of Genetics and Pathology, Rudbecklaboratoriet, Uppsala University, S-751 85 Uppsala, Sweden; 3 Department of Engineering Sciences, Uppsala University, Box 528, S-751 20 Uppsala, Sweden

## Abstract

**Background:**

Detection of periodically expressed genes from microarray data without use of known periodic and non-periodic training examples is an important problem, e.g. for identifying genes regulated by the cell-cycle in poorly characterised organisms. Commonly the investigator is only interested in genes expressed at a particular frequency that characterizes the process under study but this frequency is seldom exactly known. Previously proposed detector designs require access to labelled training examples and do not allow systematic incorporation of diffuse prior knowledge available about the period time.

**Results:**

A learning-free Bayesian detector that does not rely on labelled training examples and allows incorporation of prior knowledge about the period time is introduced. It is shown to outperform two recently proposed alternative learning-free detectors on simulated data generated with models that are different from the one used for detector design. Results from applying the detector to mRNA expression time profiles from *S. cerevisiae *showsthat the genes detected as periodically expressed only contain a small fraction of the cell-cycle genes inferred from mutant phenotype. For example, when the probability of false alarm was equal to 7%, only 12% of the cell-cycle genes were detected. The genes detected as periodically expressed were found to have a statistically significant overrepresentation of known cell-cycle regulated sequence motifs. One known sequence motif and 18 putative motifs, previously not associated with periodic expression, were also over represented.

**Conclusion:**

In comparison with recently proposed alternative learning-free detectors for periodic gene expression, Bayesian inference allows systematic incorporation of diffuse *a priori *knowledge about, e.g. the period time. This results in relative performance improvements due to increased robustness against errors in the underlying assumptions. Results from applying the detector to mRNA expression time profiles from *S. cerevisiae *include several new findings that deserve further experimental studies.

## Background

Several different algorithms for detection of periodically expressed genes in DNA microarray temporal profiles have been proposed [1-7]. Theoretical and algorithmic foundations for the detection algorithms include for example Fourier analysis [1,2], spline modelling [6], single-pulse models [5], and partial least squares classification [7]. One group of algorithms, including those in [1,3,5,6], use supervised learning methods [8] that exploit labelled expression profiles of genes known to be periodically expressed in the experiment to find other genes that also are periodic. This supervised learning approach precludes many potential applications where labelled training examples are not available e.g. for poorly characterised organisms.

In the subgroup of recently proposed learning-free algorithms which do not rely on supervised learning, prior knowledge in the form of a known angular frequency ω is presumed. For example, in [2] the power (amplitude) of frequency ω in the expression profile Fourier spectrum is used in creating a score for detection. However, since the period time usually is not exactly known, a novel method was proposed in [4] to resolve this problem by employing Fisher's g-test [8] for detection of periodic temporal profiles. This approach seems attractive but is designed to detect any periodic temporal profile, even if its period differs significantly from the period of the cell-cycle. In other words, it does not use any prior knowledge about the period time.

The algorithm used in the work by Spellman *et al*. (1998) [1] partly utilises prior knowledge about the cell-cycle period. This is achieved by averaging the power of Fourier spectrum frequencies across a discrete set of frequencies over a frequency interval that is thought to encompass the true frequency (corresponding to the period time as determined by auxiliary experimental techniques). The detector test statistic is modulated by the Pearson correlations between the present temporal profile and a set of labelled temporal profiles for genes known to be periodically expressed. Thus, the algorithm belongs to the subgroup of supervised methods.

The performance of various algorithms used for detection of cell-cycle genes, *i.e*. genes regulated by and/or regulating the cell-cycle, were recently compared by de Lichtenberg *et al*. (2005) [2]. Importantly, one should remember that not all genes, required for the cell-cycle are periodically expressed. One example is cdc28 kinase whose activity is phase specific but regulated by the cyclins on the protein level [9]. Moreover, there are many periodically expressed genes that are not part of the cell-cycle machinery. For example, the gene encoding the cell wall mannoprotein TIR1 (ORF id YER011W) is known to be periodically expressed (it is included in set B1 of de Lichtenberg *et al*. [2]). In the Gene Ontology [10] TIR1 is annotated to the biological process of stress-response, but not to any annotation linked to the cell cycle. With this in mind, inferred periodic expression from temporal patterns of mRNA expression may be used to study to what degree cell-cycle genes are periodically expressed, but it is not expected that a detector for periodic expression will recover all cell-cycle genes.

Any detector for periodic transcription transforms the expression time profile into a real number, the test statistic, which is used to make a decision or to rank the genes. The sensitivity and specificity of the detector thus depends on the particular test statistic threshold employed and should therefore be evaluated for all relevant threshold settings in a Receiver Operating Characteristic (ROC) analysis. The ROC is usually displayed in the form of a curve that shows the probability of detection as a function of the probability of false alarm for all possible values of the threshold [11]. Due to the small number of labelled temporal gene expression profiles presently available, it is not possible to perform reliable evaluation of the performance of the detectors on real biological data. For budding yeast *S. cerevisiae *there are compilations of genes known to be periodically expressed during the cell-cycle [2] but there are no compilations of genes known to be non-periodic. Thus, using the few genes known to be periodically expressed, a rough estimate of the probability of detection can be obtained for any threshold value but there will be no estimates at all of the probability of false alarm.

In addition to the lack of labelled data for performance validation, another difficulty is that a gene may very well be periodically expressed in one experimental condition but not in another [12]. In conclusion, meaningful evaluations of detectors must be performed on simulated data. Since the exact distribution of the temporal profiles of genes that are periodically expressed in any experiment is not known, it is important to study the robustness of the method when it is applied to time profiles generated from models different from those assumed in the construction of the detector.

In this work we demonstrate how Bayesian inference [13,14] can use diffuse prior knowledge about the cell-cycle period time to achieve improved detection of periodically expressed genes with a period close to that of the cell-cycle, without help from any labelled training examples provided by a supervisor. In a recently proposed empirical Bayesian approach to detection of periodically expressed genes [3], Bayesian inference is used to compensate for undesirable phase de-synchronization among the cells in the population during the cell-cycle experiments. However, Bayesian inference is not used in the critical step of designing a likelihood ratio based optimal detector that maximizes the probability of detection *P*_*D *_for a given choice of the probability of false alarm *P*_*FA*_. The new Bayesian detector we introduce here calculates an approximation of the desired likelihood ratio and is based on diffuse but prior knowledge about the cell-cycle period time that has not been employed in earlier approaches.

We compare the performance of the Bayesian detector introduced here to two state-of-the-art algorithms for learning-free detection of periodically expressed genes. The first is a detector based on Fisher's g-test [4]. It is the only detector proposed for this purpose that is learning-free without requiring the period time to be exactly known. The second detector is based on a combination of two separate score values. It requires the period time to be exactly known and has performed well in a recently reported study [2].

## Results

### Evaluation on simulated data

Simulated periodic gene expressions were generated by adding random noise drawn from a normal distribution to fixed periodic temporal waveforms (sinus, saw-tooth, square). Simulated non-periodic gene expressions were generated by samples from another normal distribution. We do not believe this choice of waveforms to correspond to naturally occurring time profiles. However, using several different waveforms provides information about the robustness of the detectors against model errors. In addition to the periodic waveforms, we also simulated gene expression profiles that were periodic with a multiplicative amplitude attenuation similar to what has been observed in experimental data [1,15].

Comparisons of the Bayesian detector to Fisher's g-test [4,8] and a combination test proposed by de Lichtenberg *et al*. [2] were performed. In Fisher's g-test, the sampling distribution of the magnitude *I*(*ω*_*k*_) of the strongest Fourier spectrum component *ω*_*k *_in samples from a sequence of independent, identically distributed Gaussian distributions (white noise), is used in a classical hypothesis test. Specifically, the statistic

g=max⁡kI(ωk)∑k=1[N2]I(ωk)
 MathType@MTEF@5@5@+=feaafiart1ev1aaatCvAUfKttLearuWrP9MDH5MBPbIqV92AaeXatLxBI9gBaebbnrfifHhDYfgasaacH8akY=wiFfYdH8Gipec8Eeeu0xXdbba9frFj0=OqFfea0dXdd9vqai=hGuQ8kuc9pgc9s8qqaq=dirpe0xb9q8qiLsFr0=vr0=vr0dc8meaabaqaciaacaGaaeqabaqabeGadaaakeaacqWGNbWzcqGH9aqpdaWcaaqaamaaxababaGagiyBa0MaeiyyaeMaeiiEaGhaleaacqWGRbWAaeqaaOGaemysaKKaeiikaGccciGae8xYdC3aaSbaaSqaaiabdUgaRbqabaGccqGGPaqkaeaadaaeWbqaaiabdMeajjabcIcaOiab=L8a3naaBaaaleaacqWGRbWAaeqaaOGaeiykaKcaleaacqWGRbWAcqGH9aqpcqaIXaqmaeaadaWadaqaamaaliaabaGaemOta4eabaGaeGOmaidaaaGaay5waiaaw2faaaqdcqGHris5aaaaaaa@4AF3@

is calculated for each gene where *N *is the number of time points and *ω*_*k *_*= 2π k/N*. The sampling distribution of this statistic for time-series from a white noise Gaussian process has been determined analytically and is used in a hypothesis test.

In the approach by de Lichtenberg *et al*. [2], expression profiles are ranked by the combination test statistic

F=Ptotal⋅[1+(Pregulation0.001)2]⋅[1+(Pperiodicity0.001)2]
 MathType@MTEF@5@5@+=feaafiart1ev1aaatCvAUfKttLearuWrP9MDH5MBPbIqV92AaeXatLxBI9gBaebbnrfifHhDYfgasaacH8akY=wiFfYdH8Gipec8Eeeu0xXdbba9frFj0=OqFfea0dXdd9vqai=hGuQ8kuc9pgc9s8qqaq=dirpe0xb9q8qiLsFr0=vr0=vr0dc8meaabaqaciaacaGaaeqabaqabeGadaaakeaacqWGgbGrcqGH9aqpcqWGqbaudaWgaaWcbaGaemiDaqNaem4Ba8MaemiDaqNaemyyaeMaemiBaWgabeaakiabgwSixpaadmaabaGaeGymaeJaey4kaSYaaeWaaeaadaWcaaqaaiabdcfaqnaaBaaaleaacqWGYbGCcqWGLbqzcqWGNbWzcqWG1bqDcqWGSbaBcqWGHbqycqWG0baDcqWGPbqAcqWGVbWBcqWGUbGBaeqaaaGcbaGaeGimaaJaeiOla4IaeGimaaJaeGimaaJaeGymaedaaaGaayjkaiaawMcaamaaCaaaleqabaGaeGOmaidaaaGccaGLBbGaayzxaaGaeyyXIC9aamWaaeaacqaIXaqmcqGHRaWkdaqadaqaamaalaaabaGaemiuaa1aaSbaaSqaaiabdchaWjabdwgaLjabdkhaYjabdMgaPjabd+gaVjabdsgaKjabdMgaPjabdogaJjabdMgaPjabdsha0jabdMha5bqabaaakeaacqaIWaamcqGGUaGlcqaIWaamcqaIWaamcqaIXaqmaaaacaGLOaGaayzkaaWaaWbaaSqabeaacqaIYaGmaaaakiaawUfacaGLDbaaaaa@71B0@

that depends on two p-values obtained via resampling, *P*_*regulation *_and *P*_*periodicity*_. *P*_*regulation *_is intended to reflect the probability that the standard deviation of a particular gene can be obtained by pure chance. *P*_*periodicity *_is intended to reflect the chance of obtaining a random signal with more energy in the *user defined *frequency component (period time) of interest than the corresponding energy in the particular gene time profile of interest. *P*_*total *_is defined as the product *P*_*periodicity*_·*P*_*regulation*_.

**Figure 1:**
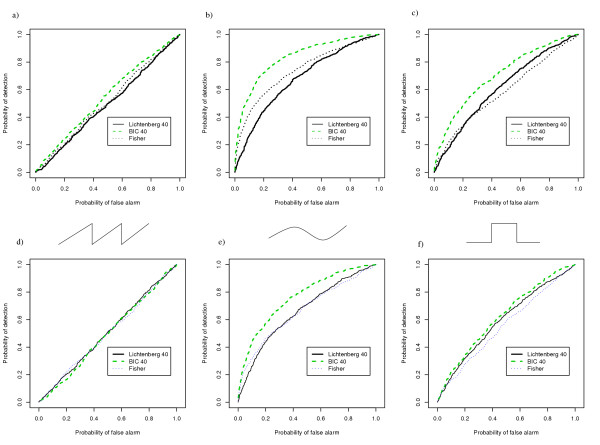
**ROC curves for simulated data with different wave forms. **The Bayesian detector (BIC 40) with a Gaussian prior with mean 40 and standard deviation 0.1, the Fisher's g-test detector (Fisher), and the combination test detector (Lichtenberg) for the period time 40 min, were all applied to 1000 samples each from a set of simulated periodic signals and non-periodic signals. The periodic signals had period 50 min and amplitude 1, sampled at 5 min intervals over two periods (100 min). Gaussian white noise with standard deviation 1.0 was added. The set of non-periodic signals was formed by sampling a Gaussian distribution with standard deviation equal to the standard deviation of the periodic class and mean zero. Results are shown for three different waveforms, sawtooth (a), sinusoid (b) and square (c). As can be expected the detectors perform best on the sinusoidal waveform (b). The relatively larger robustness of the Bayesian detector is clearly revealed as it outperforms the other two detectors in all three cases. We also studied the effects of attenuation by multiplying each of the waveforms with an exponentially decreasing factor e^-αt ^(d, e, f). The attenuation coefficient α of the exponential was chosen such that the amplitude at 100 min was 70% of that at 0 min. As is seen this modification has little effect on the performance of the detectors, but naturally its performance will degrade more with faster attenuation.

Figure 1 shows ROC curves for the three detectors studied using simulated data for three different waveforms (sinusoid, sawtooth and square). The data emulates typical microarray experiment sampling procedures with 20 samples spaced 5 mins apart encompassing two periods (100 min for 50 min period signal). In this case, we assumed that the estimated period time was 40 min and that the Bayesian and the combination detector were designed based on this information with the Bayesian detector also using a diffuse prior that reflects uncertainty about the period time estimate. The Bayesian detector used a Gaussian prior with standard deviation of 0.1 and a mean of 2π /T, where T is indicated in the figure legends. Clearly, the Bayesian detector outperformed both Fisher's g-test and the combination test in all instances and appears to be more robust than the other detectors since performance was better on all three waveforms. As can also been seen in Figure 1 all detectors have poor performance on the sawtooth waveform. This clearly demonstrates the limitations of detectors based on a single sinusoidal frequency. It should be noted that the signal-to-noise ratio used in the simulations corresponds to a relatively high level of experimental noise and that all detectors performs much better when the noise level is decreased (data not shown).

It has been observed that the time profiles of yeast gene expression in synchronized populations exhibit attenuation [1,15]. In order to study the effect of this attenuation, we also evaluated the result of including a multiplicative exponential term e^-α*t *^to the simulated time profiles from the periodic class. The attenutation coefficient α was chosen such that that the amplitude of expression at 100 min was 70% of that at 0 min. The attenuation coefficient α was selected to reflect the rate of attenuation that has been observed in microarray time profiles of periodically expressed genes in *S. cerevisiae *[1,15]. As seen in Figure 1, the Bayesian detector performs well compared to the other two detectors also on these generative models. The performances are of course expected to become worse for all detectors with growing values of α (faster attenuation).

**Figure 2:**
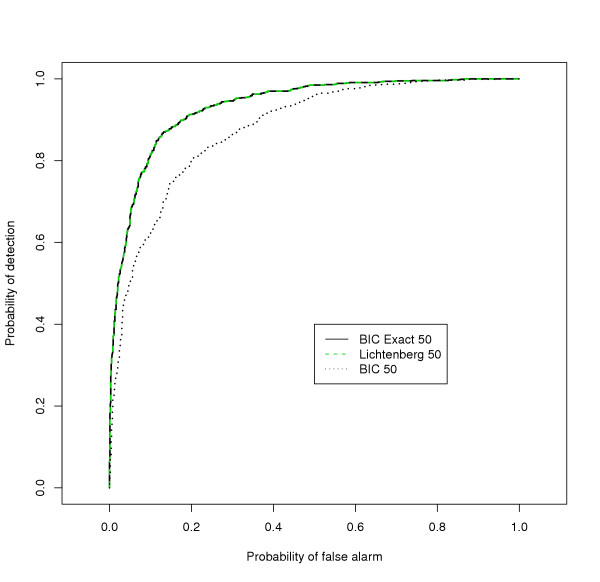
**Impact of certainty in prior information. **Using the same settings as those used to generate Figure 1 we analysed the performance of the combination test and Bayesian detectors for the correct period time of 50 min. When the standard deviation of period time prior of the Bayesian detector was set to zero, the combination test detector (Lichtenberg) and the Bayesian detector (BIC Exact 50) perform almost identically. Also shown is the performance of the Bayesian detector when the standard deviation was changed to 0.1(BIC 50).

Exact knowledge of the period time results in a sharp prior in the Bayesian detector (a Dirac delta function located at the correct angular velocity). As shown in Figure 2, in this case the combination test and the Bayesian detector have essentially the same performance. The ROC for the Bayesian detector when the standard deviation of the prior has been increased to 0.1 is also presented in Figure 2. Now the performance drops but is still not far from the performance obtained when the period time is exactly known.

We also evaluated the performance of the Bayesian detector when the simulated noise was non-gaussian (Laplace and uniform) and obtained in similar results (data not shown). Since the Bayesian detector performance is more robust against the exact waveform than the other detectors, it is expected to work better also on real data.

### Periodically expressed genes in *S. cerevisiae*

**Figure 3:**
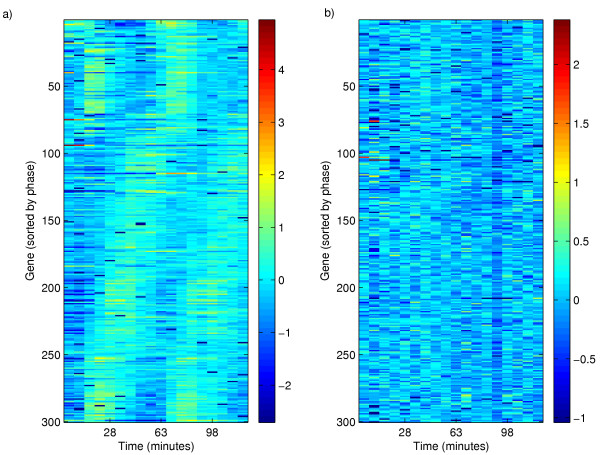
**Visualisation of time profiles from the α-factor experiment. **a) Time profiles of the 300 ORFs with the largest support for periodicity. The experiment covers roughly two cell cycles and a global periodic pattern with two peaks is clearly observed. b) Time profiles of the 300 ORFs with the least support for periodicity. No time dependency may be discerned. The genes in each plot were ordered by the maximum *a posteriori *estimate of the phase angle θ.

The Bayesian detector was applied to three time-course experiments where mRNA expression was measured using DNA microarrays during the cell-cycle in *S. cerevisiae *[1]. In these experiments, yeast cells had been synchronized by a method based on either α-factor (estimated period 55–77 min), cdc15 (estimated period 60–80 min) or cdc28 (estimated period 80–100 min). The Bayesian prior for period time was chosen so that the reported interval of *frequencies *encompassed 70% of the probability mass in a Gaussian distribution, with its mean value equal to the average of the reported frequency interval. For illustrative purposes we show in Figure 3 the temporal profiles for the 300 ORFs with the largest support for periodicity in the α-factor experiments as well as the corresponding 300 genes with the least support. The time profiles have been sorted by the maximum *a posteriori *value of the phase angle.

#### Detection of cell-cycle annotated genes

As discussed in the introduction, it is well known that some genes involved in the cell-cycle regulation are periodically expressed as well as some that are known not be periodically expressed. To what extent genes involved in the cell-cycle machinery are periodically expressed remains an open question. We addressed this question by detecting periodically expressed genes using the Bayesian detector and then analyzing how many cell-cycle genes were detected.

The cell-cycle genes were defined as the list of open reading frames annotated to the biological process "cell-cycle" (GO:0007049) or one of its descendants in the Gene Ontology [10]. Out of the 6178 open reading frames (ORFs) on the microarray used, 290 were annotated to "cell-cycle". Selection was stringent in that only ORFs annotated with the evidence code *inferred from mutant phenotype *(IMP) were used.

**Figure 4:**
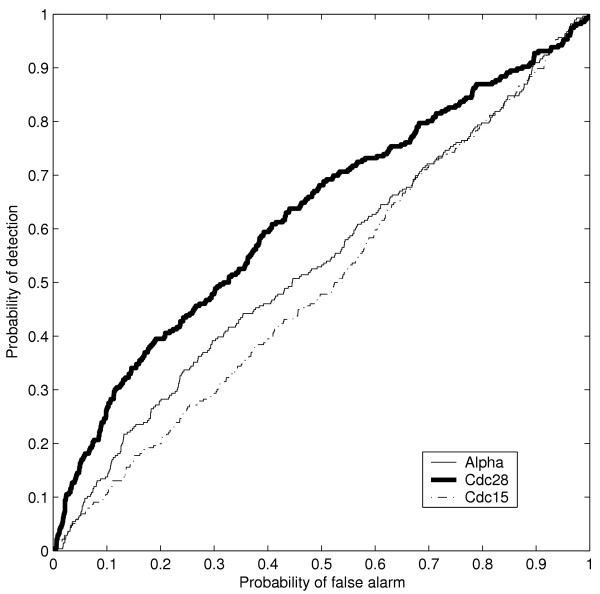
**Detection of ORFs having a "cell-cycle" annotation. **Receiver operator characteristic (ROC) curves showing the detection of ORFs annotated as "cell-cycle" (IMP). The Bayesian detector is most successful in detecting "cell cycle" genes in the *cdc28 *experiment, whereas detection in the *cdc15 *seems to occur at random.

The detector test statistic (in Equation 8, Methods section) was calculated for each ORF in each of the experiments. By varying the detection threshold, a ROC curve for each synchronization method was calculated. These ROC curves show detection of cell-cycle genes from expression data using our detector (Figure 4). As an example, for a high value of the detection threshold τ = 0.95 (corresponding to strong evidence for periodic expression) in the α-factor experiment, 452 ORFs were detected as periodic out of which 36 are annotated to cell-cycle with IMP evidence code (all ORFs detected as periodic in each experiment is available in the supplement). This corresponds to P_*D *_= 0.12 and P_*FA *_= 0.07 (see Figure 4). This result shows that there is only strong evidence for periodic expression for about 12% of the cell-cycle genes. However, the cell-cycle genes are highly overrepresented among the genes detected as periodically expressed. The probability of finding 36 (or more) out of 290 ORFs with cell-cycle IMP among 452 out of 6178 genes calculated by the hypergeometric sampling distribution is ~0.001. Similarly, finding 56 among 516 genes in the *cdc28 *experiment corresponds to a probability of ~1e-9. For lists of the ORFs with τ = 0.95 in the α-factor, *cdc28 *and *cdc15 *experiments see Additional file 1, Additional file 2 and Additional file 3.

#### Detection of genes with at least one phase-specific sequence motif

**Figure 5:**
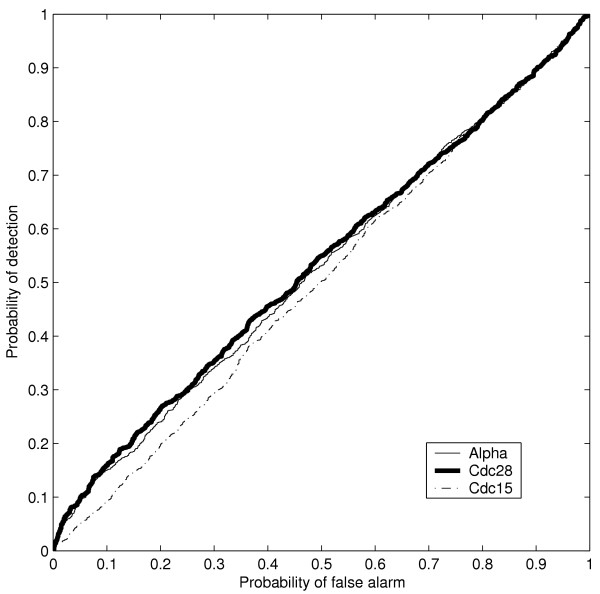
**Detection of cell-cycle motifs. **Receiver operator characteristic (ROC) curves showing detection of ORFs having any of the "cell-cycle" motifs, clearly detection rates are very low overall, indicating a low abundance of functional motifs in the compilation of sequence motifs of Hughes *et al*. [16] (see text).

We also addressed the issue of whether promoter regions of genes detected as periodically expressed are enriched with respect to cell-cycle transcription factor binding motifs. For this we used the compilation of 37 known and 319 putative sequence motifs described in [16]. Out of the 37 known motifs, seven are known to be bound by phase-specific transcription factors: MCM1, ECB, MCB, SWI5, SFF, CCA and SCB [17,18]. The compilation encompasses 5650 ORFs in the *S. cerevisiae *genome, out of which 5592 are present in the microarray dataset. There are 4390 ORFs having at least one of the seven cell-cycle motifs in their upstream region. The ROC curve reflecting the detection of this group is shown in Figure 5. For example, with τ = 0.95 (corresponding to P_*FA*_≈ 0.05 and P_*D*_≈ 0.1 for α-factor and cdc28 in Figure 5), the probability of finding at least as many genes with either one of the cell-cycle motifs as we do among the ORFs detected as periodic is 1.6e-6 (α-factor) and 7.3e-8 (cdc28). In the cdc15 experiment, ORFs with cell-cycle motifs in the upstream region are not detected more often than what could be expected at random (p-value ~0.5). Note in Figure 5 that for α-factor and cdc28, only a relatively small fraction of the genes with known cell-cycle motifs are detected as periodically expressed when the probability of false alarm is low. One explanation could be that there are additional layers of regulation that prevents the periodic expression of genes whose promoter contain cell-cycle motifs, such as epigenetic regulation. Therefore we decided to determine if such effects were equally prevalent for all known phase-specific motifs when studied individually.

#### Detection of genes containing a specific cell-cycle motif

**Figure 6:**
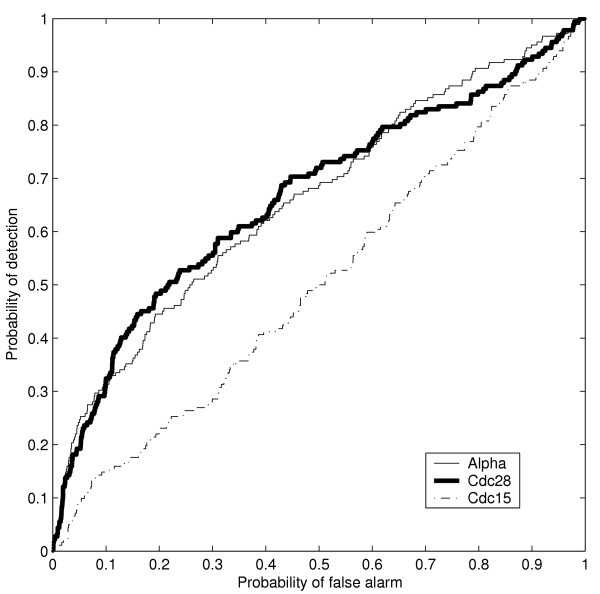
**Detection of the MCB motif. **Receiver operator characteristic (ROC) curves corresponding to detection of ORFs having the MCB motif which appears to be the motif with the largest number of functional sites (see text). Detection in the *cdc15 *experiment appears close to random though.

We investigated how often the seven known cell-cycle motifs occurred in the upstream region of genes detected as periodically expressed. Results for τ = 0.95 are shown in Table 1. None of the cell-cycle motifs were overrepresented in *cdc15 *and cell-cycle motifs CCA and SWI5 were clearly not overrepresented in upstream regions of the ORFs detected in the *cdc28 *experiment. Furthermore, for α-factor and cdc28 we noted that the detector was most successful at detecting genes with the MCB motif. Figure 6 shows the ROC curves for detection of genes with the MCB motif using the three different synchronization methods. The expression of genes with the MCB motif thus appears to be less influenced by other types of regulation than just regulation through the MCB motif, at least in the α-factor and *cdc28 *experiments.

#### Discovering novel phase-specific motifs

Since the probability of detecting a gene with at least one known cell-cycle motif is relatively low for a low rate of false alarm (Figure 5) we investigated whether this could be explained by the presence of other motifs, still unknown to have phase-specific expressions. We thus tested for overrepresented motifs in the sets of ORFs detected as periodic in each of the experiments. We found that among the 319 putative motifs, 18 motifs were significantly overrepresented among the genes detected in the α-factor experiment. The putative motif MCM1' was also overrepresented in the *cdc28 *experiment, see Table 3.

Rather surprisingly, we also found the LYS14 motif, bound by the LYS14 transcription factor and involved in regulation of genes of the lysine biosynthesis, to be significantly overrepresented among ORFs detected as periodic in the α-factor experiment.

**Table 1:** Detector performance and significance for individual motifs Detector performance and probability of overrepresentation of motifs for detection threshold τ = 0.95.

	α-factor			Cdc28		
Motif name	P_D_	P_FA_	p-value	P_D_	P_FA_	p-value

CCA	0.11	0.070	7.8e-4	0.090	0.090	0.45
ECB	0.16	0.065	5.6e-10	0.19	0.079	4.7e-10
MCB	0.26	0.065	1.1e-12	0.27	0.080	1.4e-11
MCM1	0.16	0.064	4.4e-11	0.17	0.080	5.0e-9
SCB	0.0098	0.062	6.4e-6	0.10	0.080	0.0063
SFF	0.080	0.059	0.0010	0.10	0.068	1.14e-5
SWI5	0.088	0.065	0.0016	0.088	0.086	0.42

**Table 3:** Overrepresented putative motifs in α-factor experiment Only motifs significant at a Bonferroni corrected 0.05 level shown. * = Not significant. Italics indicates motif also significant in *cdc28*, see the text.

Motif name	p-value
ALPHA1'	< 1e-6
SFF'	< 1e-6
m_PNDE6	3e-6
m_other_transport_facilitators_orfnum2SD_n10	1.4e-5
m_other_morphogenetic_activities_orfnum2SD_n7	< 1e-6
m_organization_of_chromosome_structure_orfnum2SD_n12	2.5e-5
m_organization_of_chromosome_structure_orfnum2SD_n20	2.4e-5
m_cytok9	1e-6
m_pheromone_response_generation_orfnum2SD_n12	2.3e-5
M_g_proteins_orfnum2SD_n12	< 1e-6
M_g_proteins_orfnum2SD_n13	2.4e-5
M_other_energy_generation_activities_orfnum2D_n12	1e-6
M_anion_transporters_orfnum2SD_n9	1e-6
M_allantoin_and_allantoate_transporters_orfnum2SD_n12	1e-6
M_deoxyribonucleotide_metabolism_orfnum2SD_n5	1.4e-6
M_cell_death_orfnum2SD_n16	2.1e-5
M_breakdown_of_lipids_fatty_acids_and_isoprenoids_orfnum2SD_n8	1e-6
*MCM1'*	< 1e-6

## Discussion

A periodic gene expression during the cell-cycle corresponds to a phase-specific expression that in turn indicates phase-specific regulation of expression. Therefore, detection of periodic expressions in cell-cycle experiments has gained a lot of attention as a computational tool for improved understanding of cell-cycle regulation. Often the investigator knows the cell-cycle period time roughly, and if not, it could be estimated experimentally. However, such estimates will in general be uncertain due to the small size of the sample sets available. In the Bayesian framework presented here, this kind of diffuse prior information about the period time can be taken into account. As shown by means of the simulations, this results in a detector which is more robust than detectors that rely on an almost exact estimate of the cell-cycle period time.

One should note that although the prior on the period time used in the simulations presented in Figure 1 is not very precise it results in superior performance in comparison with the Fisher's g-test which considers all possible periodicities. One explanation for the superior performance of the Bayesian detector on varying wave forms is due to a higher rate of false positive classifications using the g-test, since it is designed to detect all possible single periodicities. When the signals encountered contain multiple peaks in their Fourier spectra, the Bayesian detector still identifies them well, since the dominant term in the Fourier expansion is that of the desired period. However, the Fisher's g-test is designed for detection of *one and only one *sinusoidal signal with unknown period time. The test statistic used is the ratio of the maximum power in the Fourier spectrum to the sum of powers at all frequencies. Thus, for waveforms with the same fundamental frequency, the g-test statistic will decrease in magnitude if the Fourier spectra contain more peaks.

As expected from theory, the Bayesian detector also outperforms the combination test by de Lichtenberg *et al*. when the estimate of the period time deviates from the true value. This deterioration of performance might be explained by the decrease in the combination test output caused by the deviation between the estimated and true period time. When the period time estimate is correct, then the Bayesian detector and the combination test have essentially the same performance but having access to the correct period time is not a realistic possibility in most cases.

Although the present work considers detection of periodically expressed genes with a period close to that of the cell-cycle, it is important to note that the suggested Bayesian approach can easily be adjusted for design of other detectors. For example, it would be straightforward to design detectors for genes with periodic expression at a particular phase of the cell-cycle by applying a non-uniform prior on the phase angle. Furthermore, the phenomena of attenuating signals due to de-synchronization of phase in the cell population under study may be incorporated in the model. This would be an interesting direction for future work. However, we noted that the decrease in performance of the detector on sinusoidal waveforms with slow attenuation was small for a low rate of false alarm. Nevertheless we investigated time profiles of genes from set B1 of de Lichtenberg *et al*. [2] that were not detected (for high values of the detection threshold) but could not find a predominant signal of periodicity with attenuation for any of those genes. This suggests that those genes would not have been detected even if the Bayesian detector would have been modified to take attenuation into account.

The present work shows that the Bayesian detector detects more genes necessary for regulation of the cell-cycle than a random detector, but that the probability of detection is low for reasonable rates of false alarm. A trivial explanation is that some genes having a mutant cell-cycle phenotype are not periodically expressed as they are needed throughout the entire cell-cycle, or regulated otherwise. Nevertheless, our approach provides quantitative estimates as to what extent genes necessary for cell-cycle regulation are periodically expressed. As an example, for a stringent condition on periodicity, 36 out of 290 cell-cycle genes are detected as periodic in the α-factor experiment. In addition the reasonable but still relatively high level of false alarm rate may have the trivial explanation that many genes not required for the cell-cycle are also periodically expressed.

It is interesting to note the lack of strong coupling between periodic expression and cell-cycle transcription factor motifs in budding yeast. There are many genes having cell-cycle motifs in their promoter regions that are not detected as periodically expressed. This observation can be explained by complexities of gene regulation, e.g. regulation of chromatin accessibility [15,19]. It could also be expected that a large set of genes are conditionally cell-cycle regulated depending on the environment.

It has been argued that the synchronization methods used induce many side effects not related to the core functionality of the cell-cycle [12], thus it is to be expected that different genes are periodically expressed in different experiments. Our analysis supports this notion, since we found that there are a number of genes that are clearly periodically expressed in only one experiment. Expression could also be regulated by combinations of transcription factors. One study supporting this idea has shown that genes sharing pairs of motifs show more similar expression patterns than when considering genes sharing single motifs [17].

It is also interesting to note that many genes are detected as periodically expressed while their promoter regions contain no known cell-cycle regulated elements. Wolfsberg *et al*. (1999) used a set of genes determined to be periodically expressed from microarray experiments to search for novel cell-cycle sequence motifs in yeast [20]. Consistent with the present study, the presence of most known cell-cycle motifs were not well correlated with periodic expression and a similar pattern can be expected for novel motifs. However, a strong sequence signal for MCB and MCB-like motifs was found [20]. Our results also indicate that the MCB motif is the motif that is most strongly associated with periodic transcription. One interpretation is that genes regulated by the MCB motif have less complex regulation of expression than genes with other motifs. Similar observations were made by Cho *et al*. who used manual identification of periodically genes in *cdc28 *synchronized *S. cerevisiae *and studied the 500 bp upstream region of the periodically expressed genes [15]. They found that the presence of MCB and SCB motifs were associated with periodicity. However, a large number of the periodically expressed genes did not have MCB, SCB or any other of the known phase specific motifs [15].

We find that several putative sequence motifs [16] are significantly over-represented in the genes detected as periodically expressed (see Table 3). For instance, genes involved in DNA metabolism, morphogenetic activities and organization of chromosome structure contain some of these motifs. Furthermore, the putative motifs SFF' and MCM1' are associated with the known cell-cycle motifs SFF and MCM1 respectively. Thus, our findings support earlier notions that these putative motifs are important for periodic expression of cell-cycle related genes.

In addition to our simulations and experiments on real data already described, the Bayesian detector has also been applied to the biological test sets described by de Lichtenberg *et al*. [2]. Since these test sets only contain positive examples, it is important to note that they do not provide any estimate at all of the probability of false alarm which is as important as the probability of detection in a ROC analysis. Therefore the results obtained using these test sets are available only as supplemental information [see Additional file 4, Additional file 5, Additional file 6].

## Conclusion

Learning-free detectors that do not rely on a set of training examples are perhaps the only alternative when trying to identify the periodically expressed genes of poorly characterized organisms for which there is also limited knowledge available from closely related organisms. Our simulations indicate that systematic incorporation of *a priori *knowledge about the cell-cycle period time using Bayesian inference may improve detection performance in comparison with two other recently proposed learning-free detectors.

When applying our detector to real data from Spellman *et al*. [1], we found that genes detected as periodically expressed only contain a small fraction of cell-cycle genes inferred from mutant phenotype. We also found a statistical overrepresentation of cell-cycle regulated sequence motifs among the genes detected as periodically expressed. Moreover, among the genes detected, we found 19 motifs that have not previously been implicated in regulation of periodic expression in budding yeast. Their roles can now be tested by direct experiments and improve our understanding of *cis*-acting mechanisms underlying periodic transcription.

## Methods

### The detection problem

The Bayesian detector derived here should, after collection of a temporal profile consisting of samples *y*(*t*) from a time interval [*a, b*], determine whether the temporal profile contains a periodic signal with a period close to the cell-cycle time, or not. We express this as the binary hypothesis test

*H*_1 _: *y*_*t *_= *A*_0 _+ *A*_1 _cos(ω *t + θ) + u*(*t*)

*H*_0 _: *y*_*t *_= *B *+ v(*t*)    (1)

where both *u*(*t*) and *v*(*t*) are white Gaussian stochastic processes (i.e. no time dependencies) with unknown standard deviations σ_u _and σ_v_, respectively. Model *H*_*1 *_is regarded as a truncated Fourier series of the actual periodic signal. Adding higher order Fourier series terms as in [3] would be possible but it is expected that for the signals of interest to the investigator, the term containing the fundamental frequency which corresponds to the cell-cycle period time will be much larger than the rest. Support for this assumption comes from the reported success of the detectors used in [1,2] and from the results reported here. Furthermore, in typical microarray assays of reported cell-cycle experiments, a conventional principal component analysis (PCA) of the expression data covariance matrix shows that the variance in the data can be ascribed to sinusoidal periodic expression with a period time close to that of the cell-cycle [21,22].

One should note that the terms *u *and *v *in Eq. (1) may contain both measurement noise and model errors. We would also like to stress that by employing a Gaussian distribution for *u *and *v*, the detector becomes sensitive only to the first and second order moments of the observed errors *ε*_*1*_(t)=*y*_*t *_-*A*_*0*_-*A*_*1 *_*cos(ω*_*o*_*t+θ*) and *ε*_0_(*t*) = *y*_*t *_*- B *respectively. This partly explains the robustness of our approach against different distributions of measurement noise and model errors. As long as the first and second order statistics are equal for different problems, the Bayesian test statistic will stay unchanged.

In the Bayesian framework the probabilities for model *H*_1 _and *H*_0 _respectively are obtained as

P(Hi|D)=P(D|Hi)P(Hi)∑P(D|Hj)P(Hj)     (2)
 MathType@MTEF@5@5@+=feaafiart1ev1aaatCvAUfKttLearuWrP9MDH5MBPbIqV92AaeXatLxBI9gBaebbnrfifHhDYfgasaacH8akY=wiFfYdH8Gipec8Eeeu0xXdbba9frFj0=OqFfea0dXdd9vqai=hGuQ8kuc9pgc9s8qqaq=dirpe0xb9q8qiLsFr0=vr0=vr0dc8meaabaqaciaacaGaaeqabaqabeGadaaakeaacqWGqbaucqGGOaakcqWGibasdaWgaaWcbaGaemyAaKgabeaakiabcYha8jabdseaejabcMcaPiabg2da9maalaaabaGaemiuaaLaeiikaGIaemiraqKaeiiFaWNaemisaG0aaSbaaSqaaiabdMgaPbqabaGccqGGPaqkcqWGqbaucqGGOaakcqWGibasdaWgaaWcbaGaemyAaKgabeaakiabcMcaPaqaamaaqaeabaGaemiuaaLaeiikaGIaemiraqKaeiiFaWNaemisaG0aaSbaaSqaaiabdQgaQbqabaGccqGGPaqkcqWGqbaucqGGOaakcqWGibasdaWgaaWcbaGaemOAaOgabeaakiabcMcaPaWcbeqab0GaeyyeIuoaaaGccaWLjaGaaCzcamaabmaabaGaeGOmaidacaGLOaGaayzkaaaaaa@56EF@

where i = 0 or i = 1. *P*(*D*|*H*_*i*_) is the likelihood and *P*(*H*_*i*_) the prior probability of model *H*_*i*_. The likelihood is obtained by marginalization of the unknown parameters. If model *H*_*i *_is parametrized by *ψ*_*i *_then

P(D|Hi)=∫ψi∈Ψidψi p(D|ψi,Hi)p(ψi|Hi)     (3)
 MathType@MTEF@5@5@+=feaafiart1ev1aaatCvAUfKttLearuWrP9MDH5MBPbIqV92AaeXatLxBI9gBaebbnrfifHhDYfgasaacH8akY=wiFfYdH8Gipec8Eeeu0xXdbba9frFj0=OqFfea0dXdd9vqai=hGuQ8kuc9pgc9s8qqaq=dirpe0xb9q8qiLsFr0=vr0=vr0dc8meaabaqaciaacaGaaeqabaqabeGadaaakeaacqWGqbaucqGGOaakcqWGebarcqGG8baFcqWGibasdaWgaaWcbaGaemyAaKgabeaakiabcMcaPiabg2da9maapefabaacbiGae8hzaqgcciGae4hYdK3aaSbaaSqaaiab=LgaPbqabaGccaaMc8UaemiCaaNaeiikaGIaemiraqKaeiiFaWNae4hYdK3aaSbaaSqaaiabdMgaPbqabaGccqGGSaalcqWGibasdaWgaaWcbaGaemyAaKgabeaakiabcMcaPiabdchaWjabcIcaOiab+H8a5naaBaaaleaacqWGPbqAaeqaaOGaeiiFaWNaemisaG0aaSbaaSqaaiabdMgaPbqabaGccqGGPaqkaSqaaiab+H8a5naaBaaameaacqWGPbqAaeqaaSGaeyicI4SaeuiQdK1aaSbaaWqaaiabdMgaPbqabaaaleqaniabgUIiYdGccaWLjaGaaCzcamaabmaabaGaeG4mamdacaGLOaGaayzkaaaaaa@614F@

where *P*(*ψ*_*i *_|*H*_*i*_) reflects the prior information about the parameters. Here we assume that the investigator has no prior information about the parameters except for the period time *T *= 2π /ω. Furthermore, we assume the parameters are independent. Hence the prior distribution factors with respect to the parameters; for model *H*_0 _we have p(*B*, *σ*_*v*_) = p(*B*)p(*σ*_*v*_), and for *H*_1 _we get p(*A*_0_, *A*_1_, *ω*, *θ*, *σ*_*u*_) = p(*A*_0_)p(*A*_1_)p(*ω*)p(*θ*)p(*σ*_*u*_). As described before, the prior p(*ω*) is chosen to reflect the knowledge the investigator has about the cell-cycle period time *T*. If it is known for certain that *T *is in the interval [*T*_1_, *T*_2_], a uniform prior on this interval is suitable. In the present work we focus on the situation when the investigator knows the expected mean and variance of the prior distribution of *ω *and we therefore use a Gaussian prior distribution for *ω *(the maximum entropy distribution). The hyperparameters of p(*ω*), the mean *μ*_ω _and standard deviation *σ*_ω _must thus be chosen to reflect the prior knowledge of the investigator. As mentioned earlier, the prior for the cell-cycle period time is chosen so that the reported interval of corresponding *frequencies *encompassed 70% of the probability mass in a Gaussian distribution, with its mean value equal to the average of the reported frequency interval

The priors for the remaining parameters are chosen to reflect the lack of information, thus (improper) uniform priors are used for *B *and *A*_*i*_. The prior for *θ *is uniform on [*0, π*]. For the standard deviations *σ*_*i *_we use Jeffrey's prior which is suitable due to its invariance to scaling of the variables involved [14]. Thus the resulting integrals (see Eq. 3) to be computed become

P(D|H1)=∫⋯∫ψi∈ΨidσudωdθdA0dA112πσuN+1σωexp⁡(−12σu2∑(yn−(A0+A1cos⁡(ωtn+θ))2−(ω−μω)22σω2)     (4)
 MathType@MTEF@5@5@+=feaafiart1ev1aaatCvAUfKttLearuWrP9MDH5MBPbIqV92AaeXatLxBI9gBaebbnrfifHhDYfgasaacH8akY=wiFfYdH8Gipec8Eeeu0xXdbba9frFj0=OqFfea0dXdd9vqai=hGuQ8kuc9pgc9s8qqaq=dirpe0xb9q8qiLsFr0=vr0=vr0dc8meaabaqaciaacaGaaeqabaqabeGadaaakeaafaqadeGabaaabaGaemiuaaLaeiikaGIaemiraqKaeiiFaWNaemisaG0aaSbaaSqaaiabigdaXaqabaGccqGGPaqkcqGH9aqpdaWdbaqaaiabl+UimnaapefabaGaemizaqgcciGae83Wdm3aaSbaaSqaaiabdwha1bqabaGccqWGKbazcqWFjpWDcqWGKbazcqWF4oqCcqWGKbazcqWGbbqqdaWgaaWcbaGaeGimaadabeaakiabdsgaKjabdgeabnaaBaaaleaacqaIXaqmaeqaaaqaaiab=H8a5naaBaaameaacqWGPbqAaeqaaSGaeyicI4SaeuiQdK1aaSbaaWqaaiabdMgaPbqabaaaleqaniabgUIiYdaaleqabeqdcqGHRiI8aaGcbaWaaSaaaeaacqaIXaqmaeaadaGcaaqaaiabikdaYiab=b8aWbWcbeaakiab=n8aZnaaDaaaleaacqWG1bqDaeaacqWGobGtcqGHRaWkcqaIXaqmaaGccqWFdpWCdaWgaaWcbaGae8xYdChabeaaaaGccyGGLbqzcqGG4baEcqGGWbaCdaqadaqaaiabgkHiTmaalaaabaGaeGymaedabaGaeGOmaiJae83Wdm3aa0baaSqaaiabdwha1bqaaiabikdaYaaaaaGcdaaeabqaaiabcIcaOiabdMha5naaBaaaleaacqWGUbGBaeqaaOGaeyOeI0IaeiikaGIaemyqae0aaSbaaSqaaiabicdaWaqabaGccqGHRaWkcqWGbbqqdaWgaaWcbaGaeGymaedabeaakiGbcogaJjabc+gaVjabcohaZjabcIcaOiab=L8a3jabdsha0naaBaaaleaacqWGUbGBaeqaaOGaey4kaSIae8hUdeNaeiykaKIaeiykaKYaaWbaaSqabeaacqaIYaGmaaaabeqab0GaeyyeIuoakiabgkHiTmaalaaabaGaeiikaGIae8xYdCNaeyOeI0Iae8hVd02aaSbaaSqaaiab=L8a3bqabaGccqGGPaqkdaahaaWcbeqaaiabikdaYaaaaOqaaiabikdaYiab=n8aZnaaDaaaleaacqWFjpWDaeaacqaIYaGmaaaaaaGccaGLOaGaayzkaaaaaiaaxMaacaWLjaWaaeWaaeaacqaI0aanaiaawIcacaGLPaaaaaa@A035@

for model H_1 _and

P(D|H0)=∫∫dσvdB12πσuN+1exp⁡(−12σu2∑(yn−B)2)     (5)
 MathType@MTEF@5@5@+=feaafiart1ev1aaatCvAUfKttLearuWrP9MDH5MBPbIqV92AaeXatLxBI9gBaebbnrfifHhDYfgasaacH8akY=wiFfYdH8Gipec8Eeeu0xXdbba9frFj0=OqFfea0dXdd9vqai=hGuQ8kuc9pgc9s8qqaq=dirpe0xb9q8qiLsFr0=vr0=vr0dc8meaabaqaciaacaGaaeqabaqabeGadaaakeaacqWGqbaucqGGOaakcqWGebarcqGG8baFcqWGibasdaWgaaWcbaGaeGimaadabeaakiabcMcaPiabg2da9maapeaabaWaa8qaaeaacqWGKbaziiGacqWFdpWCdaWgaaWcbaGaemODayhabeaakiabdsgaKjabdkeacbWcbeqab0Gaey4kIipakmaalaaabaGaeGymaedabaWaaOaaaeaacqaIYaGmcqWFapaCaSqabaGccqWFdpWCdaqhaaWcbaGaemyDauhabaGaemOta4Kaey4kaSIaeGymaedaaaaakiGbcwgaLjabcIha4jabcchaWnaabmaabaGaeyOeI0YaaSaaaeaacqaIXaqmaeaacqaIYaGmcqWFdpWCdaqhaaWcbaGaemyDauhabaGaeGOmaidaaaaakmaaqaeabaGaeiikaGIaemyEaK3aaSbaaSqaaiabd6gaUbqabaGccqGHsislcqWGcbGqcqGGPaqkdaahaaWcbeqaaiabikdaYaaaaeqabeqdcqGHris5aaGccaGLOaGaayzkaaaaleqabeqdcqGHRiI8aOGaaCzcaiaaxMaadaqadaqaaiabiwda1aGaayjkaiaawMcaaaaa@6566@

for model H_0_. The last double integral is analytically tractable but the former integral over the five parameters is not [23]. To avoid time consuming numerical integration of that integral, we are using the standard Bayesian Information Criterion (BIC) approximation [13] which is defined as:

BIC(Hi)≡ln⁡P(D|ψiMAP,Hi)−d2ln⁡N≈ln⁡P(D|Hi)     (6)
 MathType@MTEF@5@5@+=feaafiart1ev1aaatCvAUfKttLearuWrP9MDH5MBPbIqV92AaeXatLxBI9gBaebbnrfifHhDYfgasaacH8akY=wiFfYdH8Gipec8Eeeu0xXdbba9frFj0=OqFfea0dXdd9vqai=hGuQ8kuc9pgc9s8qqaq=dirpe0xb9q8qiLsFr0=vr0=vr0dc8meaabaqaciaacaGaaeqabaqabeGadaaakeaacqWGcbGqcqWGjbqscqWGdbWqcqGGOaakcqWGibasdaWgaaWcbaGaemyAaKgabeaakiabcMcaPiabggMi6kGbcYgaSjabc6gaUjabdcfaqjabcIcaOiabdseaejabcYha8HGaciab=H8a5naaDaaaleaacqWGPbqAaeaacqWGnbqtcqWGbbqqcqWGqbauaaGccqGGSaalcqWGibasdaWgaaWcbaGaemyAaKgabeaakiabcMcaPiabgkHiTmaalaaabaGaemizaqgabaGaeGOmaidaaiGbcYgaSjabc6gaUjabd6eaojabgIKi7kGbcYgaSjabc6gaUjabdcfaqjabcIcaOiabdseaejabcYha8jabdIeainaaBaaaleaacqWGPbqAaeqaaOGaeiykaKIaaCzcaiaaxMaadaqadaqaaiabiAda2aGaayjkaiaawMcaaaaa@6001@

where superscript *MAP *indicates that the log likelihood should be evaluated at the maximum a posteriori (MAP) parameter setting. *d *is the number of parameters in model *H*_*i *_and *N *the number of samples available. Now the log odds of model *H*_*1 *_over *H*_*0*, _*log(P(H*_1_*|D)/P(H*_*0*_*|D))*, may be approximated as *BIC(H*_1_*)- BIC(H*_0_*) *- *τ *where *τ= P*(*H*_*i*_)/*P*(H_*0*_). Since the detector assigns a gene as periodic when this approximation is above some predefined threshold *τ *', the detection rule is to assign a gene as periodic if *BIC(H*_1_*)- BIC(H*_0_*) *- *τ *> *τ*'. Equivalently, assign a gene as periodic if

*g(D) = BIC(H*_1_*)- BIC(H*_0_*) *> *τ *   (7)

where *g(D) *is the detector test statistic and *τ *now is a redefined arbitrary detector threshold. Also, note that the test statistic *g(D) *in (7) is an approximation of the log-likelihood ratio which should be used to maximize *P*_*D *_for a given *P*_*FA *_(the Neyman-Pearson criterion) [24]. The output of the detector is a function of the time (t) and gene expression (y) measurements. Finally, in order to obtain a test statistic that is bounded to the interval [0, 1], the value of *g(D) *was converted into a probability by the monotonous transformation

P(H1|D)≈s=eg(D)1+eg(D).     (8)
 MathType@MTEF@5@5@+=feaafiart1ev1aaatCvAUfKttLearuWrP9MDH5MBPbIqV92AaeXatLxBI9gBaebbnrfifHhDYfgasaacH8akY=wiFfYdH8Gipec8Eeeu0xXdbba9frFj0=OqFfea0dXdd9vqai=hGuQ8kuc9pgc9s8qqaq=dirpe0xb9q8qiLsFr0=vr0=vr0dc8meaabaqaciaacaGaaeqabaqabeGadaaakeaacqWGqbaucqGGOaakcqWGibasdaWgaaWcbaGaeGymaedabeaakiabcYha8jabdseaejabcMcaPiabgIKi7kabdohaZjabg2da9maalaaabaGaemyzau2aaWbaaSqabeaacqWGNbWzcqGGOaakcqWGebarcqGGPaqkaaaakeaacqaIXaqmcqGHRaWkcqWGLbqzdaahaaWcbeqaaiabdEgaNjabcIcaOiabdseaejabcMcaPaaaaaGccqGGUaGlcaWLjaGaaCzcamaabmaabaGaeGioaGdacaGLOaGaayzkaaaaaa@4A56@

The detector was applied to the simulated and real data. Although the test statistic is an approximation of the log likelihood ratio, as the simulation results demonstrate, it is still powerful for detection. Choice of detection threshold is arbitrary and must be decided by the investigator. Classical p-values for this test statistic may always be generated by means of resampling techniques but this is not a topic covered in this work.

### Implementation

The detector was implemented in the R language statistical processing environment () and is available upon request from the authors. The algorithm simply involves locating the MAP estimates for the models (the integrands of Eq. 4 and 5) and the evaluation of Eq 8. For *H*_*0*_, an analytical solution is used. For *H*_*1 *_a numerical approximation was found by optimisation using the Nelder-Mead simplex method [25]. The initial conditions were chosen such that the standard deviation *σ*_*u *_and *A*_*0 *_were equal to the sample standard deviation and mean of the expression measurement. The mean of the prior distribution of *ω *was used as its initial value. The initial values of *A*_*1 *_and *θ *were determined from the data by an analytical least squares fit of the model *A*_*1 *_*cos(ω*_*o*_*t+θ*) to the time series where *ω*_*o *_is the initial value of *ω*.

### Receiver operator characteristics

The Receiver Operator Characteristic (ROC) has been developed by the signal detection community to study how the probability of detection (*P*_*D*_) varies with the probability of false alarm (*P*_*FA*_) [24] and is also commonly used in medical decision making [11]. For each selected value of the decision threshold τ∈ [0, 1], we obtain different pairs of values(*P*_*FA*_, *P*_*D*_). A ROC graph displays a curve indicating these pairs as τ is changed continuously. Note that if the detector is inferring signal (hypothesis *H*_*1*_) at random with probability *P*_*D*_, the false alarms will occur at a rate *P*_*D *_resulting in *P*_*FA *_= *P*_*D*_. Thus, a random detector will yield a ROC curve consisting of a diagonal line.

## Authors' contributions

CRA and MGG derived the Bayesian detector. CRA implemented the software and performed the experiments. AI analysed the results and supervised the study together with MGG. All authors wrote, read, and approved the paper.

**Table 2:** Overrepresented known motifs Only motifs significant at a Bonferroni corrected 0.05 level shown. * = Not significant. Note that LYS14 is *not *a known cell-cycle motif.

Motif name	p-value α-factor	p-value cdc28
CCA	1e-5	*
ECB	< 1e-6	< 1e-6
MCB	< 1e-6	< 1e-6
MCM1	< 1e-6	< 1e-6
SCB	< 1e-6	*
SFF	*	1.1e-5
*LYS14*	< 1e-6	*

## Supplementary Material

Additional File 1 **Detected genes in the α-factor experiment **List of the ORFs detected as periodic in the alpha-factor experiment studied, i.e. the ORFs for which τ = 0.95. This is an ASCII file, each line containing the ORF identifier, terminated by newline. Click here for file

Additional File 2 **Detected genes in the cdc28 experiment **As Additional file 1 but for the cdc28 experiment. Click here for file

Additional File 3 **Detected genes in the cdc15 experiment **As Additional file 2 but for the cdc28 experiment. Click here for file

Additional File 4 **Results from the de Lichtenberg test sets, α-factor **Results, experiment wise, for application to the B1, B2 and B3 test sets of de Lichtenberg *et al*, "Comparison of computational methods for the identification of cell cycle regulated genes", Bioinformatics (2005). The fraction of the test set detected vs the number of ORFs from the top of the ranking list of periodicity is plotted for each of the experiments. It is important to note when comparing results to those presented in by de Lichtenberg (ibid) that no period time fitted to the expression profiles of genes known (presumed) to be periodically expressed where used. Naturally such a fit will improve detection of those genes. Click here for file

Additional File 5 **Results from the de Lichtenberg test sets, cdc28 **As Additional file 4 but for the cdc28 experiment. Click here for file

Additional File 6 **Results from the de Lichtenberg test sets, cdc15 **As Additional file 4 but for the cdc15 experiment. Click here for file
