# Supplementary figures and images for: Bayesian detection of periodic mRNA time profiles without use of training examples
Source: BMC Bioinformatics. 2006 Feb 9;7:63. doi: 10.1186/1471-2105-7-63 (PMC1413563; doi:10.1186/1471-2105-7-63)

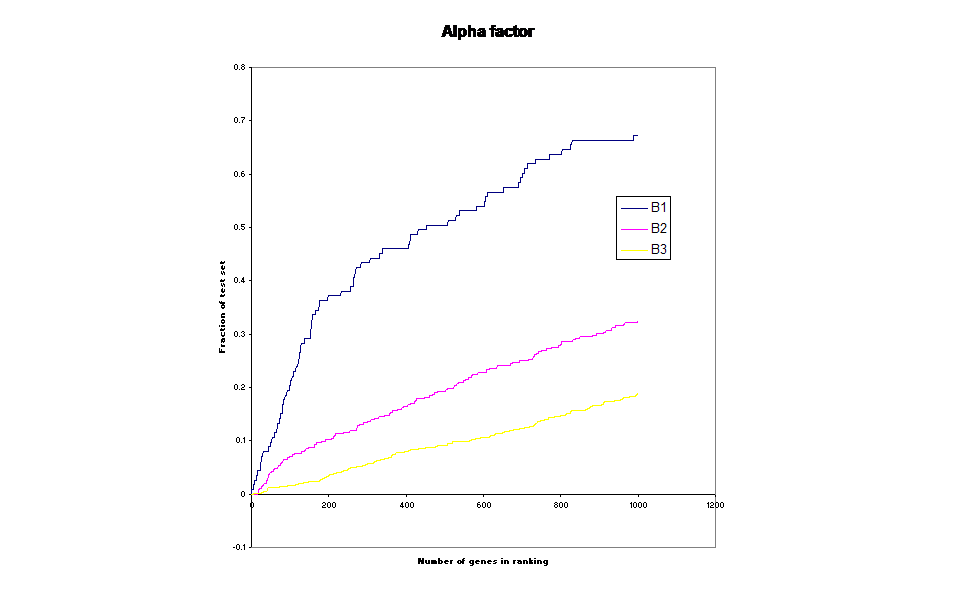

Supplement: Additional File 4 — Results from the de Lichtenberg test sets, α-factor Results, experiment wise, for application to the B1, B2 and B3 test sets of de Lichtenberg et al, "Comparison of computational methods for the identification of cell cycle regulated genes", Bioinformatics (2005). The fraction of the test set detected vs the number of ORFs from the top of the ranking list of periodicity is plotted for each of the experiments. It is important to note when comparing results to those presented in by de Lichtenberg (ibid) that no period time fitted to the expression profiles of genes known (presumed) to be periodically expressed where used. Naturally such a fit will improve detection of those genes. [file 1471-2105-7-63-S4.gif]

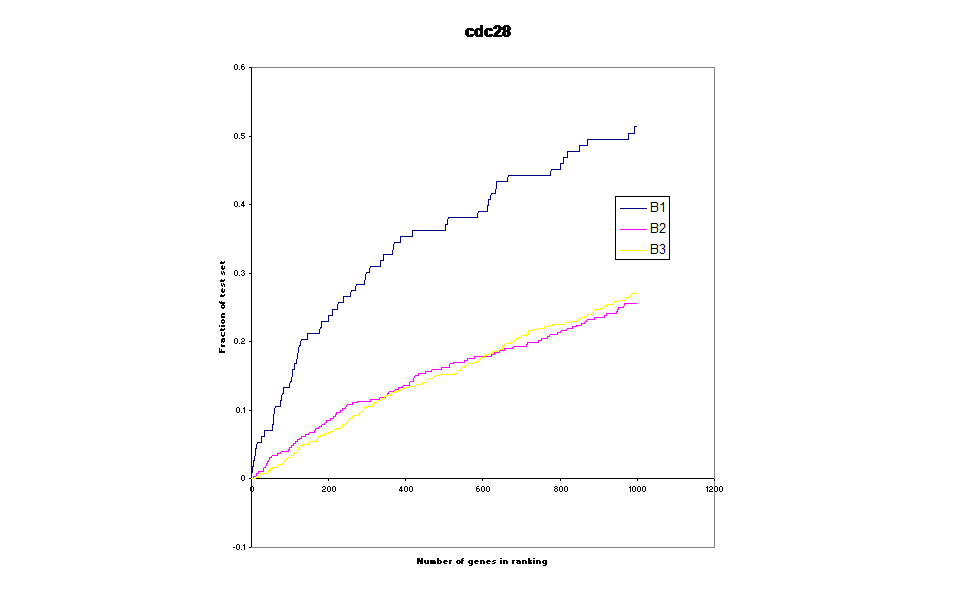

Supplement: Additional File 5 — Results from the de Lichtenberg test sets, cdc28 As Additional file 4 but for the cdc28 experiment. [file 1471-2105-7-63-S5.gif]

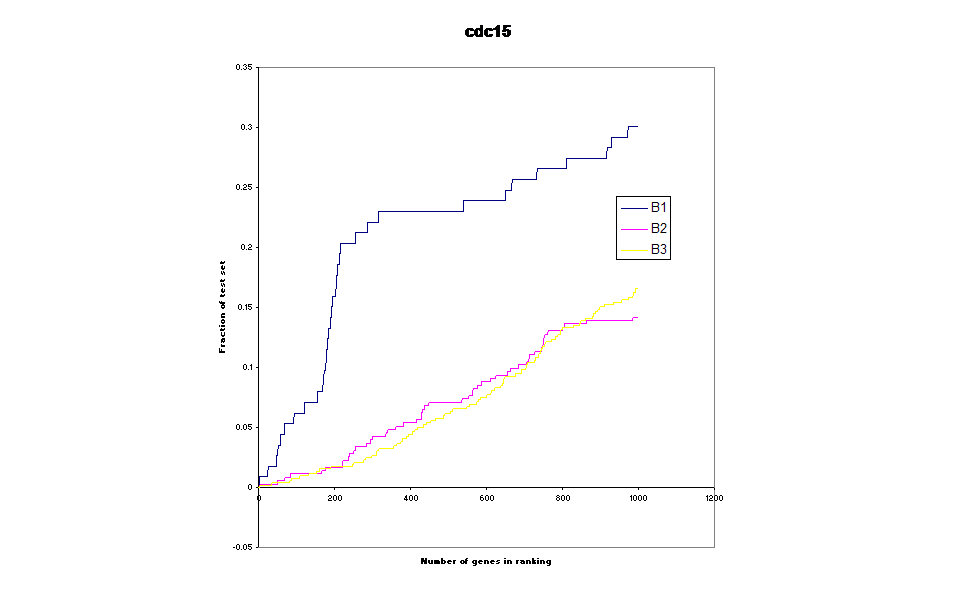

Supplement: Additional File 6 — Results from the de Lichtenberg test sets, cdc15 As Additional file 4 but for the cdc15 experiment. [file 1471-2105-7-63-S6.gif]
